# Performance of machine learning algorithms for lung cancer prediction: a comparative approach

**DOI:** 10.1038/s41598-024-58345-8

**Published:** 2024-08-09

**Authors:** Satya Prakash Maurya, Pushpendra Singh Sisodia, Rahul Mishra, Devesh Pratap singh

**Affiliations:** 1 Department of Computer Science and Engineering, Graphic Era (Deemed to be University), Dehradun, India; 2 Department of Computer Science and Engineering, Indus University, Ahmedabad, 382115 India; 3 Department of Electronics and Computer Engineering, National Institute of Advanced Manufacturing Technology (NIAMT), Ranchi, India

**Keywords:** Lung cancer, Machine learning, Classification, Prediction, Confusion matrix, Heat map correlation, Health care, Engineering, Mathematics and computing

## Abstract

Due to the excessive growth of PM 2.5 in aerosol, the cases of lung cancer are increasing rapidly and are most severe among other types as the highest mortality rate. In most of the cases, lung cancer is detected with least symptoms at its later stage. Hence, clinical records may play a vital role to diagnose this disease at the correct stage for suitable medication to cure it. To detect lung cancer an accurate prediction method is needed which is significantly reliable. In the digital clinical record era with advancement in computing algorithms including machine learning techniques opens an opportunity to ease the process. Various machine learning algorithms may be applied over realistic clinical data but the predictive power is yet to be comprehended for accurate results. This paper envisages to compare twelve potential machine learning algorithms over clinical data with eleven symptoms of lung cancer along with two major habits of patients to predict a positive case accurately. The result has been found based on classification and heat map correlation. K-Nearest Neighbor Model and Bernoulli Naive Bayes Model are found most significant methods for early lung cancer prediction.

## Introduction

The respiratory disease has enormously increased over the last decades which may be directly associated with the exposer of humans to the polluted atmosphere. Sustainable development goals (SDGs) ensure an aspiration of health and well-being for all^1^, target 3.9 is associated with reducing death and illness from air, water, and soil pollution^2^. Lung cancer is one of the most lethal diseases caused with increasing mortality rates globally by air pollution. Usually, this type of cancer begins in the lungs and may spread to other section of the body and its causes includes smoking, air pollution, and exposure to peculiar chemicals^3^. The prognosis for lung cancer varies depending on the type, stage, and overall health of the individual. The initial phases of lung cancer may not usually manifest symptoms. If early symptoms manifest, they may encompass symptoms such as short breathing, in addition to unforeseen symptoms like back pain. Tumors can lead to back pain by exerting pressure on the lungs or by spreading to the patient’s spinal cord and ribs^4^. Additional initial symptoms of lung cancer may encompass: a persistent or getting worse cough, expectorating phlegm or blood, exacerbation of chest pain during deep breathing, laughter, or coughing, hoarseness, wheezing, weakness, and fatigue, reduced appetite and weight loss, recurring respiratory infections like pneumonia or bronchitis^5^. The initial manifestations of lung cancer may be subtle, however, an early diagnosis is crucial for effective treatment alternatives and potential results.

However, it is a great challenge to detect and diagnose it in the early stage by doctors and researchers. The advancement in the storage of health records on digital platforms and data visualizations improved pattern analysis^6^. The early prediction of disease based on symptoms and textual information may enhance the diagnosis system. Aside from medical methods, soft computing techniques like applying machine learning algorithms to the main features of large, complicated lung cancer datasets may be significant for a specialist to find the disease early. On the contrary, the precision of detection depends on the availability of data and the process of selecting important measures, which further results in adequate treatment decisions.

Diverse mathematical models have already been utilized for the detection and prevention of diseases to facilitate early treatment. However, if lung cancer is diagnosed three years after its onset, it becomes unpreventable, and the likelihood of survival is extremely poor^7,8^. Nevertheless, it is possible to treat the disease when the earliest signs are present before metastasis. Thus, if cancer is found within a specific time-frame of curability, along with various risk factors for further diagnosis, a suitable therapy can be provided to the patient, enabling the implementation of appropriate preventive measures. Several computer methods have been used to find or predict lung cancer, which helps doctors figure out the best way to treat patients and their chances of survival after being diagnosed. Researchers in the field of medical sciences have employed machine learning and soft computing approaches to accurately diagnose several forms of cancer in their early stages using categorization methods. Furthermore, researchers have identified various cutting-edge methods for early-stage prognosis of cancer therapy outcomes^9^. However, it is crucial to determine an appropriate learning algorithm for the purpose of detecting lung cancer and its correlation with the patient’s habits. This research aims to conduct a comparative analysis of several machine learning algorithms on the characteristics related to lung cancer, specifically focusing on the symptoms exhibited by patients and their habits.

## Machine learning algorithms in lung cancer prediction

Lung cancer also referred to as lung carcinoma in the usual medical term, is originally a malignant tumor that grows in lung cells uncontrollably and can be identified by cell proliferation. Recent advancements in computer vision have enabled scientists to introduce various diagnostic methods using temporal image analysis^10^. However, with the growth in clinical data repositories, not only image analysis but also text data played a vital role in diagnosis. Several lung cancer studies focus on detection using symptom data and treatment decisions based on artificial intelligence, image processing, and learning algorithms. Several researchers implied neural network, support vector machine and decision tree^10^ convolutional neural network based non-linear cellular automata^11^ Random Forest, XGBoost, and Logistic Regression^12^ i.e. machine learning algorithms on clinical dataset to predict the recurrence of lung cancer and its survivability. A few comparative studies have also been presented such as ensemble techniques of Bagging and Adaboost and K-Nearest Neighbors, Decision Tree, and Neural Networks on Surveillance, Epidemiology and End Results (SEER) dataset^13^, XGBoost, GridSearchCV, Logistic Regression, Support Vector Machine, Gaussian Naïve Bayes, Decision tree, and K-Nearest Neighbor classifiers^14^ to evaluate lung cancer prediction through precision, recall, F1-Score parameters generated using confusion matrix and Area Under Curve (AUC) & Receiver Operating Characteristic (ROC) analysis. A few more machine learning classifiers such as Logistic Regression, Naïve Bayes and Random Forest, Support Vector Machine (SVM), Artificial Neural Network (ANN), k-Nearest Neighbors (KNN), Radial Basis Function Network (RBF), J48, MLP, Gradient Boosted Tree, Majority Voting, also tried for observing the performance of lung cancer prediction^15,16^. Specifically, some standard machine learning techniques such as decision tree, boosting, random forest, neural network, naïve bayes, KNN, SVM are frequent in lung cancer prediction^17^. These machine learning algorithms showed their applicability on a temporal real-world larger dataset of lung cancer for risk prediction^18,19^.

In binary classification, while using various methods, especially in diagnostic, prognostic and predictive research, Receiver Operating Characteristic (ROC) and Area under the Curve (AUC) analysis is an effective technique usually utilized to calculate measurement for the assessment of the differentiating ability of methods^20^. The ROC curve is used to assess a test’s overall diagnostic performance and compare the performance of two or more diagnostic tests^21^. In other words, the ROC is informative about the performance over a series of thresholds and can be summarized by the AUC, which is a single number^22^. Also, A gender and age based study for a lung cancer dataset has been performed using machine learning which shows the potential of applicability of naïve bayes, SVM, KNN, random forest, decision tree, AdaboostM1, and neural network^23^.

Apart from the above analysis, it is essential to inter-relate patient’s habits and symptoms, hence more precise in diagnosing and treating lung cancer. Moreover, it is equally important to find a suitable method of analyzing these datasets. Very few attempts have been made to compare different machine-learning methods for lung cancer prediction.

## Dataset preparation and analysis

Dataset for lung cancer prediction has been collected from the source The dataset consists of a total of 16 attributes with 310 instances. The dataset attributes in the given instances are distributed over gender i.e., male and female. Table 1 illustrates the detailed description of all the 16 input feature attributes in the lung cancer study dataset, which are used in the prediction of lung cancer. The attributes are divided into two categories Habits and Symptoms, which may take values as positive or negative where represented by numeric 2 [yes] and 1 [no] respectively Table 2. There were thirty-three duplicate entries among the given instances in this dataset, which were removed before processing. The instance frequency count was performed, and positive case distribution was analyzed gender-wise. Further, the frequency analysis gender-wise has been performed for patient’s habits and the symptoms identified individually. A Pearson’s Correlation has also been plotted as a heat map to assess the attribute’s importance among the others. The attributes of the clinical dataset have been chosen based on the experts of this specialization and to measure the effectiveness of the cancer prediction system, which further helps the patient to know their cancer risk with low cost and decisions based on their appropriate treatment. Data are split into two sets of (80%) for training and testing (20%) of the dataset. During the training process, each model underwent 10-fold cross validation. This involved splitting the training set into a training subset and a validation subset with a ratio of 10:1 to fine-tune the attributes. The final accuracy metric was established by using the outcomes from the ten cross-validated models and the Area Under Curve (AUC) the Receiver Operating Curve (ROC).

**Table 1:** Description of all 16 input attributes in lung cancer study dataset.

Attribute	Description [values]	Values
Gender	Indicates gender of the patient	M [Male], F [Female]
Age	Age of patients	Numeric value
Smoking	Smoking habit of patient	2 [Yes], 1 [No]
Yellow_fingers	Patient has symptom of yellow finger	2 [Yes], 1 [No]
Anxiety	Patient having anxiety	2 [Yes], 1 [No]
Peer_pressure	Patient undergoes peer pressure	2 [Yes], 1 [No]
Chronic disease	Any chronic diseases identified	2 [Yes], 1 [No]
Fatigue	Patient having fatigue	2 [Yes], 1 [No]
Allergy	Patient facing any allergy	2 [Yes], 1 [No]
Wheezing	Breathing with a husky or whistling sound	2 [Yes], 1 [No]
Alcohol consuming	Patient is alcoholic	2 [Yes], 1 [No]
Coughing	Patient having cough problem	2 [Yes], 1 [No]
Shortness of breath	Patient facing shortness of breath	2 [Yes], 1 [No]
Swallowing difficulty	Patient having difficulty in swallowing	2 [Yes], 1 [No]
Chest pain	Patient having cough problem	2 [Yes], 1 [No]
Lung_cancer	Lung cancer detected in patient	Yes[Positive], No [Negative]

**Table 2:** List of patient’s habits and symptoms in lung cancer study dataset.

Types	Attributes	Values and description
Habits	Smoking	1 [No], 2 [Yes]
	Alcohol consuming	
Symptoms	Yellow fingers	
	Anxiety	
	Peer pressure	
	Fatigue	
	Chronic diseases	
	Allergy	
	Wheezing	
	Chest pain	
	Cough	
	Shortness of breath	
	Swallowing difficulty	

## Results and discussion

**Figure 1:**
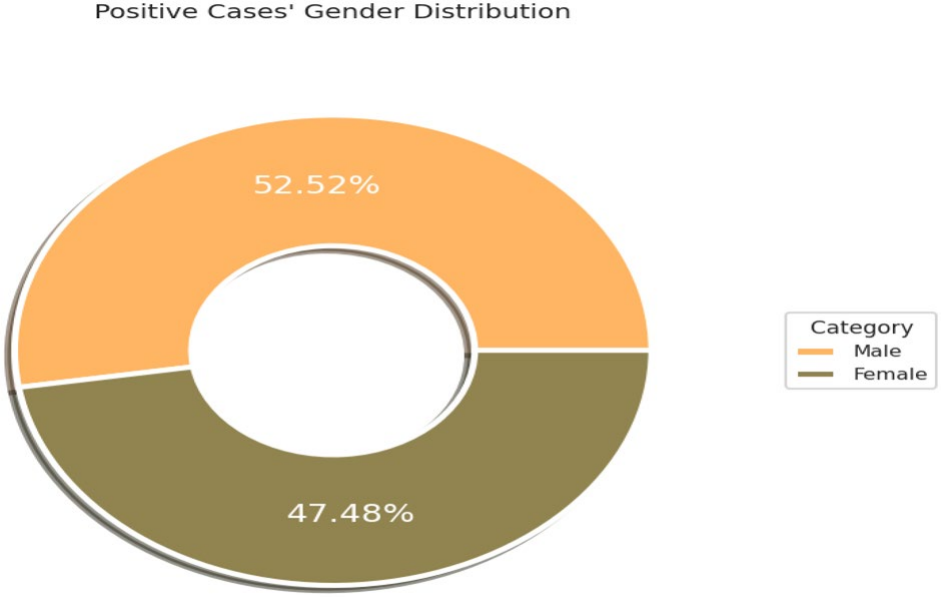
Positive case distribution gender-wise.

**Figure 2:**
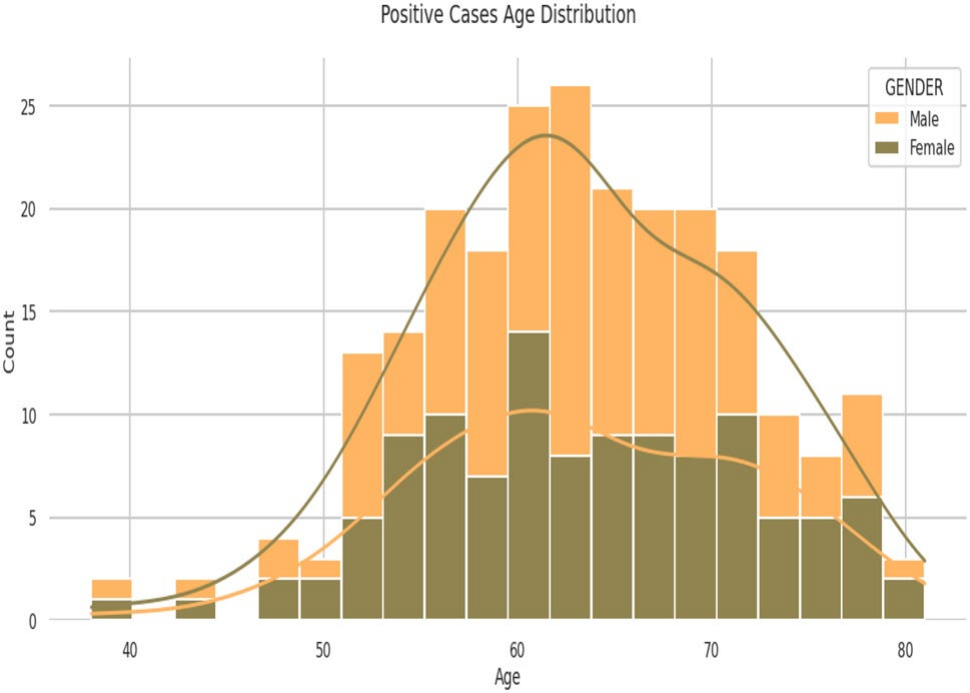
Positive case distribution age-wise over gender in the given dataset.

Primarily, the data has been analyzed based on positive and negative cases among males and females over the age distribution; Fig. 1 shows 52.52% of males and 47.48% of females are affected with the disease, while Fig. 2 shows most of the distribution identified within age 55 years and 75 years.

**Figure 3:**
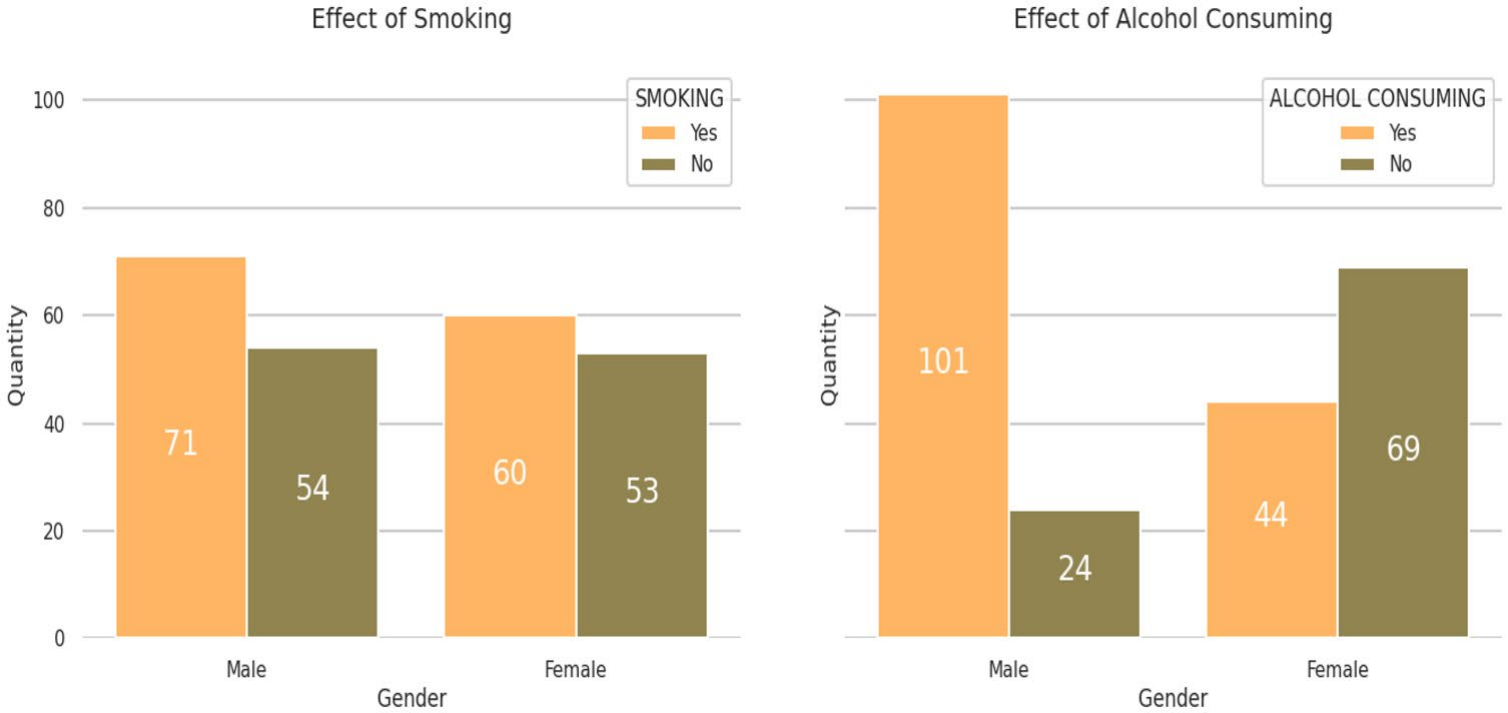
Positive and negative case distribution gender-wise over patient’s habits.

Thereafter, the next observation considered for distribution over positive and negative samples of patient’s habits, i.e., Smoking and Alcohol consumption, in which 54.2% male and 45.80% females are found to have positive cases of smoking, whereas 69.65% males and 30.35% females are found positive for alcohol consumption. Figure 3 illustrates the result for the positive and negative case distribution gender-wise over patient’s habits.

**Figure 4:**
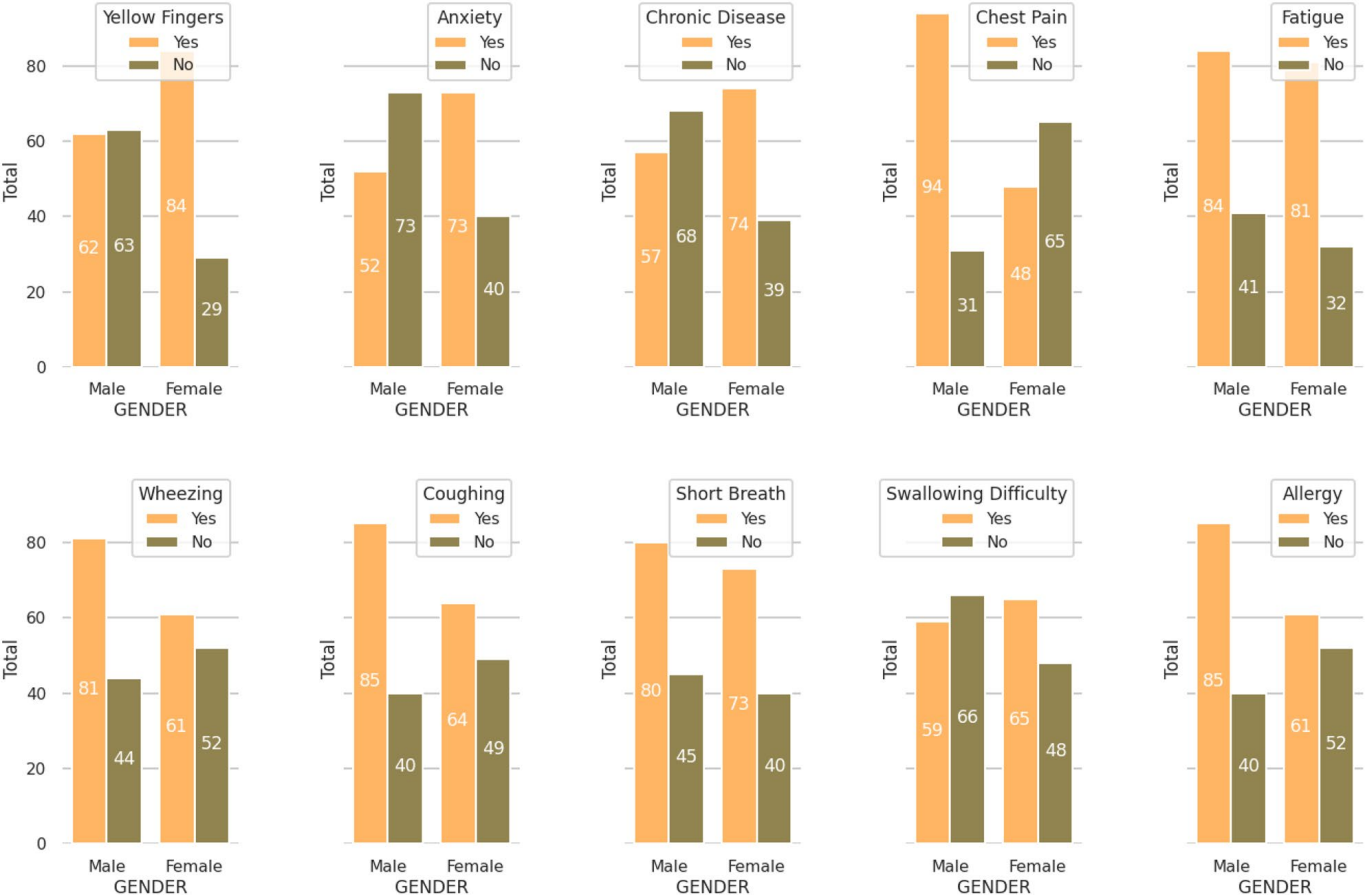
Distribution of positive and negative cases gender-wise over patient’s symptoms.

The third observation is based on the patient’s symptoms, i.e., yellow fingers, anxiety, chronic disease, chest pain, fatigue, wheezing, coughing, short breathing, swallowing difficulty, and allergy. The comprehensive study shows that 42.5% males and 57.5% females have yellow fingers, 41.6% males and 50.4% females have anxiety, 43.5% males and 56.5% females have a chronic disease, 66.2% males and 33.8% females having chest pain, 50.9% male and 49.1% female having fatigue, 57% male and 43% female having wheezing, 57% male and 43 female having coughing, 52.3% male and 47.7% female having short breath, 46.6% male and 52.4% female having swallowing, 58.2% male and 41.8% female having allergy are found positive. Figure 4 illustrates the result for the distribution of positive and negative cases gender-wise over patient’s symptoms.

**Figure 5:**
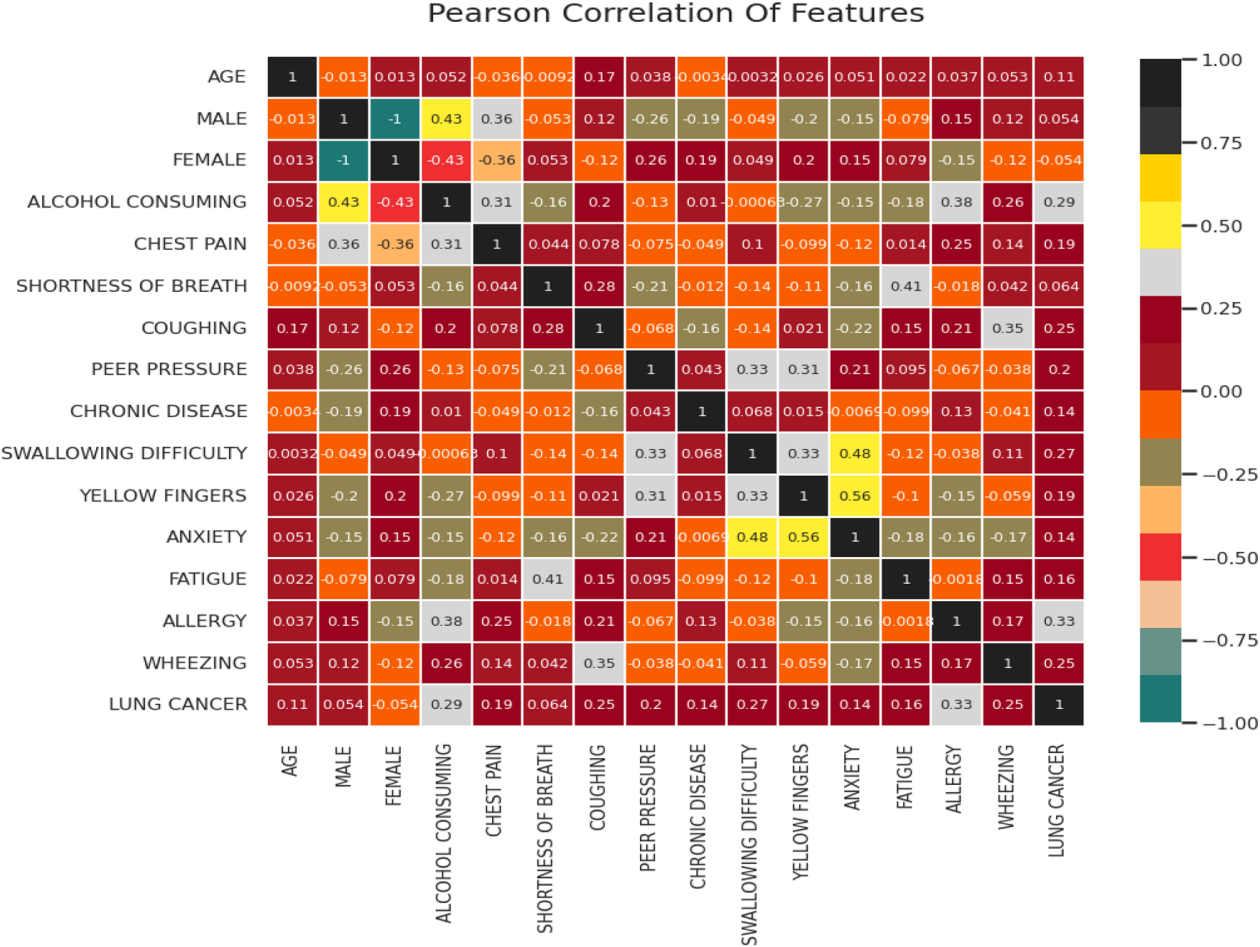
Correlation heat map for attributes considering alcohol consuming as habit of patient.

This observation shows that Yellow Finger, Coughing, Chronic Disease, Chest Pain, and Allergy are critical symptoms while studying data gender-wise. However, to know the significance of each attribute against another attribute, we have comprehended Pearson’s correlation; since alcohol consumption showed a significant effect (69.65%) on lung cancer detection, so we perform a correlation considering alcohol consumption. To analyze the correlation, the thumb rule has been directly used as Pearson correlation coefficient (r) value if $r > 0.5$ Strong Positive, $0.3< r >0.5$ is Moderate Positive, and $0 < r > 0.3$ Weak Positive. Lung cancer with alcohol-consuming habits is moderately correlated; also, it is correlated with chest pain and allergy, as shown in the correlation heat map in Fig. 5.

Now, we can apply different machine learning algorithms to understand the significance of the algorithm in this problem domain. Based on the literature survey, we have identified a few learning algorithms for lung cancer prediction viz. (1) Logistic regression, (2) Gaussian Naïve Bayes, (3) Bernoulli Naïve Bayes, (4) Support vector machine, (5) Random forest, (6) K-Nearest neighbor, (7) Extreme Gradient boosting, (8) Extra tree, (9) Ada boost, (10) Ensemble_1 with XGB and ADA, (11) Ensemble_2 with Voting Classifier, (12) Multilayer Perceptron (MLP).

### Comparison of performance of algorithms

**Figure 6:**
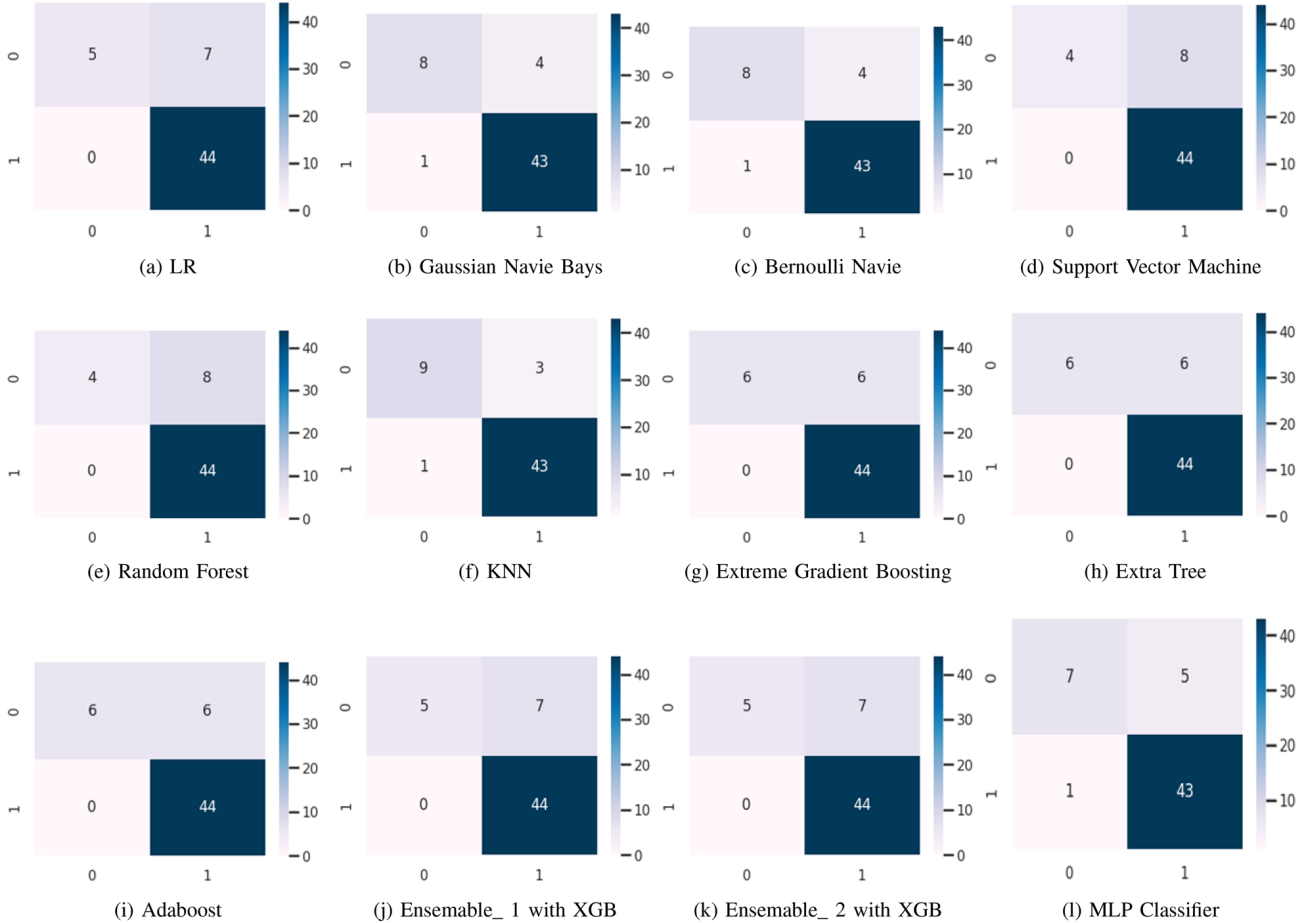
A comparative study of learning algorithm through confusion matrix over lung cancer dataset.

**Figure 7:**
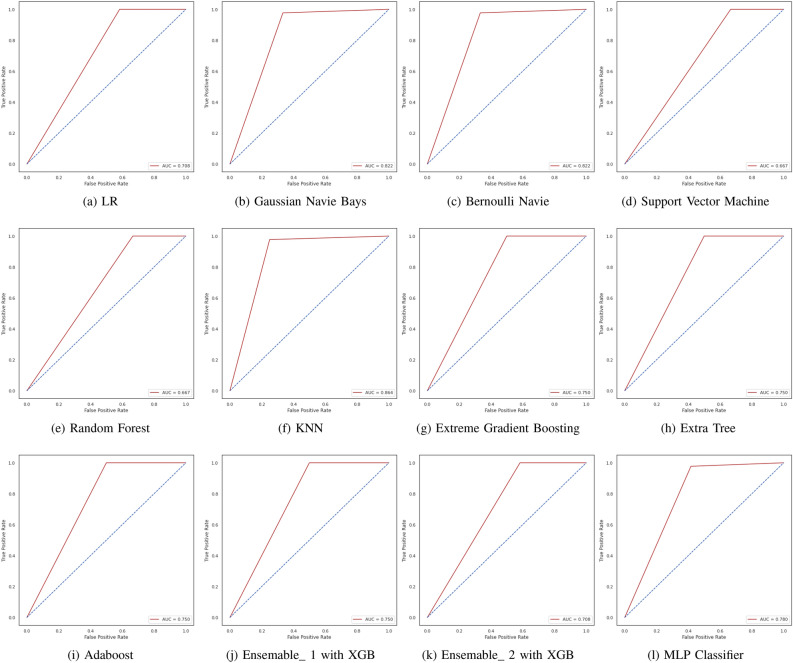
A comparative study of learning algorithm through ROC/AUC over lung cancer dataset.

We have applied different machine learning algorithms to the clinical dataset of lung cancer after preliminary statistical analysis. Based on the correlation analysis of the attributes, the dataset has been squeezed for the model for lung cancer prediction. However, to evaluate the performance of the learning algorithms confusion matrix, ROC curve (receiver operating characteristic curve) and AUC (area under the ROC curve) have been considered, whereas a detailed classification report has also been availed with each method. The models have been analyzed in Jupyter v7.0.6 run environment with the support of Python v3.11; the confusion matrix, ROC/AUC, and classification report consist of precision, recall, F1-score, and support, which are used to calculate the accuracy of the model. The analysis has been arranged in three parts viz. (1) Confusion Matrix, (2) ROC/AUC, and (3) Classification Report in which the confusion matrix is helpful to calculate precision and recall, and so the F1-score, AUC ensures the reliability of the model, and the classification report gives the overall statistics of the models. Figure 6 illustrates the confusion matrix for all the discussed machine learning models, which compares all mentioned machine learning algorithms on the confusion matrix. Figure 7 illustrates the AUC graph for all the discussed machine learning models, which compares all mentioned machine learning algorithms on the confusion matrix. Also, the set of Tables 3, 4, 5, 6, 7, 8, 9, 10, 11, 12, 13 and 14 represent the classification report for the accuracy of all the compared machine learning algorithms for lung cancer prediction.

**Table 3:** Classification report for LR classifiers.

	Precision	Recall	f1-score	Support
0	1.00	0.42	0.59	12
1	0.86	1.00	0.93	44
Macro avg	0.93	0.71	0.76	56
Weighted avg	0.89	0.88	0.85	56
Accuracy			0.88	56

The accuracy of logistic regression is 87.5%.

**Table 4:** Classification report for Gaussian Naive Bayes classifiers.

	Precision	Recall	f1-score	Support
0	0.89	0.67	0.76	12
1	0.91	0.98	0.95	44
Macro avg	0.90	0.82	0.85	56
Weighted avg	0.91	0.91	0.91	56
Accuracy			0.91	56

The accuracy of Gaussian Naive Bayes is 91.07%.

**Table 5:** Classification report for Bernaulli Navie classifier.

	Precision	Recall	f1-score	Support
0	0.89	0.67	0.76	12
1	0.91	0.98	0.95	44
Macro avg	0.90	0.82	0.85	56
Weighted avg	0.91	0.91	0.91	56
Accuracy			0.91	56

The accuracy of Bernoulli Naive Bayes is 91.07%.

**Table 6:** Classification report for SVM classifier.

	Precision	Recall	f1-score	Support
0	1.00	0.33	0.50	12
1	0.85	1.00	0.92	44
Macro avg	0.92	0.67	0.71	56
Weighted avg	0.88	0.86	0.83	56
Accuracy			0.86	56

The accuracy of Support Vector Machine is 85.71%.

**Table 7:** Classification report for Random Forest Classifiers.

	Precision	Recall	f1-score	support
0	1.00	0.33	0.50	12
1	0.85	1.00	0.92	44
Macro avg	0.92	0.67	0.71	56
Weighted avg	0.88	0.86	0.83	56
Accuracy			0.86	56

The accuracy of Random Forest Classifier is 85.71%.

**Table 8:** Classification report for K Nearest Neighbors Classifier.

	Precision	Recall	f1-score	Support
0	0.90	0.75	0.82	12
1	0.93	0.98	0.96	44
Macro avg	0.92	0.86	0.89	56
Weighted avg	0.93	0.93	0.93	56
Accuracy			0.93	56

The accuracy of K Nearest Neighbors Classifier is 92.86%.

**Table 9:** Classification report for Extreme Gradient Boosting Classifier.

	Precision	Recall	f1-score	Support
0	1.00	0.50	0.67	12
1	0.88	1.00	0.94	44
Macro avg	0.94	0.75	0.80	56
Weighted avg	0.91	0.89	0.88	56
Accuracy			0.89	56

The accuracy of extreme gradient boosting classifier is 89.29%.

**Table 10:** Classification report for Extra Tree Classifier.

	Precision	Recall	f1-score	Support
0	1.00	0.50	0.67	12
1	0.88	1.00	0.94	44
Macro avg	0.94	0.75	0.80	56
Weighted avg	0.91	0.89	0.88	56
Accuracy			0.89	56

The accuracy of extra tree classifier is 89.29%.

**Table 11:** Classification report for Ada Boost Classifier.

	Precision	Recall	f1-score	Support
0	1.00	0.50	0.67	12
1	0.88	1.00	0.94	44
Macro avg	0.94	0.75	0.80	56
Weighted avg	0.91	0.89	0.88	56
Accuracy			0.89	56

The accuracy of ada boost classifier is 89.29%.

**Table 12:** Classification report for Ensemble_1 with XGB and ADA Classifier.

	Precision	Recall	f1-score	Support
0	1.00	0.50	0.67	12
1	0.88	1.00	0.94	44
Macro avg	0.94	0.75	0.80	56
Weighted avg	0.91	0.89	0.88	56
Accuracy			0.89	56

The accuracy of Ensemble_1 with XGB and ADA Classifier is 89.29%.

**Table 13:** Classification report for Ensemble_2 with Voting Classifier.

	Precision	Recall	f1-score	Support
0	1.00	0.42	0.59	12
1	0.86	1.00	0.93	44
Macro avg	0.93	0.71	0.76	56
Weighted avg	0.89	0.88	0.85	56
Accuracy			0.88	56

The accuracy of Ensemble_2 with Voting Classifier is 87.5%.

**Table 14:** Classification report for MLP Classifier.

	Precision	Recall	f1-score	Support
0	0.88	0.58	0.70	12
1	0.90	0.98	0.93	44
Macro avg	0.89	0.78	0.82	56
Weighted avg	0.89	0.89	0.88	56
Accuracy			0.89	56

The accuracy of MLP Classifier is 89.29%.

The comparative study suggests that the accuracy of K-Nearest Neighbor is highest, i.e., 92.86%, and Bernoulli Naïve Bayes, Gaussian Naïve Bayes is 91.07% in Table 15. So, finally, we can conclude that the K-Nearest Neighbor and Bernoulli Naïve Bayes models give better results on the smaller dataset with binary characteristics. They are more suitable when attributes/features are highly independent in the given dataset. Since other models are dependent on correlation and training/testing splitting of the dataset, they could not be performed better for the dataset.

**Table 15:** A comparison of the accuracy of different learning algorithms applied over lung cancer.

S. No.	Model name	Accuracy (%)
1	Logistic Regression	87.5
2	Gaussian Naive Bayes	91.07
3	Bernoulli Naive Bayes	91.07
4	Support Vector Machine	85.71
5	Random Forest	85.71
6	K-Nearest Neighbors	92.86
7	Extreme Gradient Boosting	89.29
8	Extra Tree	89.29
9	ADA Boost	89.29
10	Ensemble_1 with XGB and ADA	89.29
11	Ensemble_2 with Voting Classifier	87.5
12	MLP	89.29

## Conclusion

Prediction of lung cancer can be useful if the system for cancer prediction works after symptom detection and also correlates to the patient’s habits and state about the cancer at a low risk. Furthermore, the expert may advise the suitable treatment option based on the individual’s cancer risk status. However, it is important to be precise while predicting lung cancer in a patient. The raw data having 310 instances has been processed to find positive cases gender-wise and then compared individual positive cases for each attribute gender-wise. A correlation study over alcohol consumption habits has identified that yellow finger and allergy are the main symptoms while conducting a preliminary analysis of the data. This study focused on the comprehensive analysis of twelve potential different machine learning algorithms in which the K-nearest neighbor and Bernoulli Naïve Bayes model (equally well as Gaussian Naïve Bayes) are found suitable with accuracy 92.86% and 91.07% respectively.

### Limitations and future scope

This study reveals the potentials of various machine learning algorithms on textual clinical data for lung cancer early detection. However, this study is performed on a small dataset, which depends on the patient’s habits and symptoms. The study may be performed on a larger dataset to analyse the variability in the algorithm’s performance. Moreover, a fine correlation may be established to improve the efficiency of the early-stage detection method. Moreover, This study may also be performed on a larger authentic dataset which must contain at least these 16 parameters, as the classification has been done based on symptoms and habits. Some potential algorithms such as Ensemble 1 with XGB and ADA, Multilayer Perceptron (MLP) may be further analyzed for larger dataset. Apart from statistical analysis, the study through data observation, it is likely find that males having the habit of alcohol consumption having symptom of chest pain and allergy have a higher chance of detecting lung cancer. However, we need an expert opinion for this insight, which may lead to establishing a weighting system the specific attribute in lung cancer detection.

Electronic Health Record (EHR) dataset may play a vital role in early detection of lung cancer. Thus, the clinical data may be utilized to draw the similarities among the parameters for the patient and the AUC achieved by the applied model. Further, more studies need to verify this process of model adoption.

## Data Availability

The datasets generated and/or analysed during the current study are available in the Data source: https://www.kaggle.com/datasets/sanjoli02/lung-cancer.
